# NA-CDQuant: Quantitative spectral analysis of nucleic acid structure by circular dichroism spectroscopy

**DOI:** 10.1073/pnas.2617882123

**Published:** 2026-10-06

**Authors:** Kevin Mosca, Søren Vrønning Hoffmann, Nykola C. Jones, Richard R. Sinden, Frank Wien, Sergio Marco, Véronique Arluison

**Affiliations:** ^a^ Laboratoire Léon Brillouin, UMR12 Commissariat à l’Énergie Atomique et aux énergies alternatives CNRS, Saclay, Gif-sur-Yvette 91191, France; ^b^ Synchrotron SOLEIL, DISCO beamline, L’Orme des Merisiers, Gif-sur-Yvette Saint Aubin BP48, 91192, France; ^c^Biochemistry and Biophysics mRNA Unit, Sanofi, mRNA Center of Excellence, Analytical Sciences, Marcy l’Etoile 69280, France; ^d^ ISA, Department of Physics and Astronomy, Aarhus University, Aarhus 8000, Denmark; ^e^ Department of Chemistry, Biology and Health Sciences, South Dakota School of Mines and Technology, Rapid City, SD 57701; ^f^Sanofi, Biophysics, Imaging, Particles & Process Analysis Unit Neuville, Biochemistry & Biophysics Neuville, Global Analytical Sciences, Neuville Sur Saône 69350, France; ^g^ Université Paris Cité, Unité de Formations et de Recherche des Sciences du Vivant, Paris 75006, France

**Keywords:** circular dichroism spectroscopy, spectral deconvolution, nucleic acids CD reference dataset, Nucleic acid–based therapeutics, RNA structural biology

## Abstract

Understanding the structural conformation and dynamics of nucleic acids in solution is critical for the development of nucleic acid therapeutics involving antisense or mRNA therapies, where controlling stability, efficacy, and manufacturability are important. Circular dichroism (CD) spectroscopy allows rapid and nondestructive solution analysis of the spatial arrangements of nucleic acids. While widely used to analyze protein structure, the potential of CD for DNA- and RNA-based pharmaceutical applications has remained largely unrealized because nucleic acid spectra are complex and difficult to interpret quantitatively. We introduce herein a framework (NA-CDQuant) that transforms CD from a qualitative fingerprinting method into a quantitative tool for nucleic acid structure prediction by combining spectral deconvolution with a rigorously curated synchrotron radiation circular dichroism reference library comprising 18 structurally defined conformational families. This systematic classification overcomes long-standing challenges posed by spectral overlap and structural polymorphism, enabling estimation of the fractional composition of coexisting conformations during real-time monitoring of structural transitions in solution. NA-CDQuant provides mechanistic insight into subtle conformational redistributions that may affect molecular recognition and functional performance assisting pharmaceutical development and medical application by facilitating formulation optimization, stability assessment, quality control, and regulatory support of nucleic acid–based therapeutics.

## Significance

This work establishes circular dichroism (CD) spectroscopy as a quantitative method to distinguish the conformations of DNA and RNA structures in solution. By integrating advanced spectral deconvolution with a curated library of 18 structural reference signatures, we enable estimation of the relative abundance of coexisting nucleic acid structural motifs within complex nucleic acid molecules from a single CD spectrum. The approach resolves long-standing challenges arising from structural heterogeneity and overlapping CD signals, making nucleic acid structural analysis rapid, reliable, and broadly accessible. Beyond fundamental research, this method provides a practical bridge between nucleic acid spectroscopy and functional insight, with broad implications for molecular biology and for the development and quality control of emerging nucleic acid–based medicines.

Nucleic acids (NA) are no longer viewed solely as passive carriers of genetic information. DNA and RNA adopt a rich variety of shapes in solution, far beyond the familiar double helix, and these structural rearrangements govern processes as diverse as gene regulation, translation, genome maintenance, and catalytic activity (1). Hairpins, quadruplexes, pseudoknots, and other folded motifs emerge dynamically and respond sensitively to their chemical environment. Even subtle changes in ion concentration, temperature, molecular crowding, or chemical modification can shift structural equilibria and thereby alter biological function (2–4). This structural plasticity is not only central to cell biology but also underpins the rapidly expanding field of NA–based medicines, including small interfering RNAs and mRNA therapeutics (5, 6). In these contexts, molecular shape directly influences stability, delivery, target recognition, and ultimately therapeutic performance.

Despite remarkable advances in high-resolution techniques such as X-ray crystallography, NMR spectroscopy, and cryoelectron microscopy, these methods often capture only snapshots of molecules and are constrained by sample crystallinity, concentration, or molecular size (7). In contrast, optical spectroscopies enable rapid, nondestructive measurements in solution without size limitations, making them particularly attractive for monitoring folding transitions and environmental responses. Among these methods, circular dichroism (CD) spectroscopy is especially sensitive to the spatial arrangement of NA bases. In the ultraviolet range, CD signals arise from chiral electronic interactions between stacked bases [n–π* and π–π* electronic excitations (8)], producing characteristic spectral patterns that reflect helicity, ribose chirality, base- stacking, -pairing, -tilting, -rolling, and overall secondary structure.

CD spectroscopy is widely used in pharmaceutical laboratories for protein characterization because quantitative interpretation methods are well established (9, 10). For NAs, however, the situation is fundamentally different. DNA and RNA populate heterogeneous ensembles of conformations, and their spectral signatures often overlap extensively. Canonical helices, partially folded intermediates, and noncanonical structures such as G-quadruplexes may coexist in a single sample, producing composite spectra that are difficult to interpret quantitatively (11, 12). Although Synchrotron Radiation Circular Dichroism (SRCD) extends the accessible wavelength range down to below 175 nm for water solubilized NAs and increases structural information content, the absence of a comprehensive and standardized reference framework has limited CD analysis of NAs largely to qualitative comparisons. Thus, the need for quantitative, solution-based structural assessment has become increasingly urgent with the rise of NA therapeutics. Drug performance depends not only on sequence but also on conformational equilibria that influence degradation resistance, molecular recognition, and batch-to-batch consistency. Regulatory evaluation and quality control therefore require analytical tools capable of detecting subtle structural shifts under formulation-relevant conditions. Yet, unlike proteins, NAs lack an established CD-based reference system comparable to those used for protein secondary structure estimation (13, 14). To address this gap, we developed a comprehensive spectral reference framework and an accompanying computational deconvolution strategy that translates complex CD signals into quantitative structural information. By organizing NA conformations into defined reference families and applying statistical optimization and machine learning approaches, our method enables the extraction of conformational distributions directly from experimental spectra. This advance transforms CD spectroscopy from a descriptive to a quantitative analytical technique for NA analysis. Beyond expanding the fundamental understanding of NA folding in solution, it provides a route toward integrating structure-sensitive spectroscopy into the development, optimization, and quality control of emerging NA–based therapeutics, diagnostics, and nanotechnology.

## Results

### Definition and Description of the 18 SRCD Spectral References.

Based on our previously developed workflow using Singular Value Decomposition (SVD) designed to classify circular dichroism spectra of NA (15), we analyzed 225 CD spectra representative of the main NAs folds. Precisely, we applied our workflow to an extended database containing 72 RNA spectra, 147 DNA spectra, and six hybrid structures. Most of the spectra were acquired at the DISCO beamline at Synchrotron SOLEIL (16) and at the AU-CD beamline at the ASTRID2 synchrotron (17); additional spectra were obtained from the literature and digitized from published spectra (18). We identified 18 representative references, the sequences used to establish them are reported in *SI Appendix*, Table S1. The reference spectra are unitless, as they correspond to singular vectors extracted from a Singular Value Decomposition process, only the singular vectors were used without using their corresponding singular values carrying the units. These vectors form an orthonormal basis describing the relative spectral shape and its variation along the wavelength range. Our approach includes the possibility to add additional references in the future. The additional spectra analyzed will be updated in the Nucleic Acid Circular Dichroism Database NACDDB (19).

**Fig. 1.**
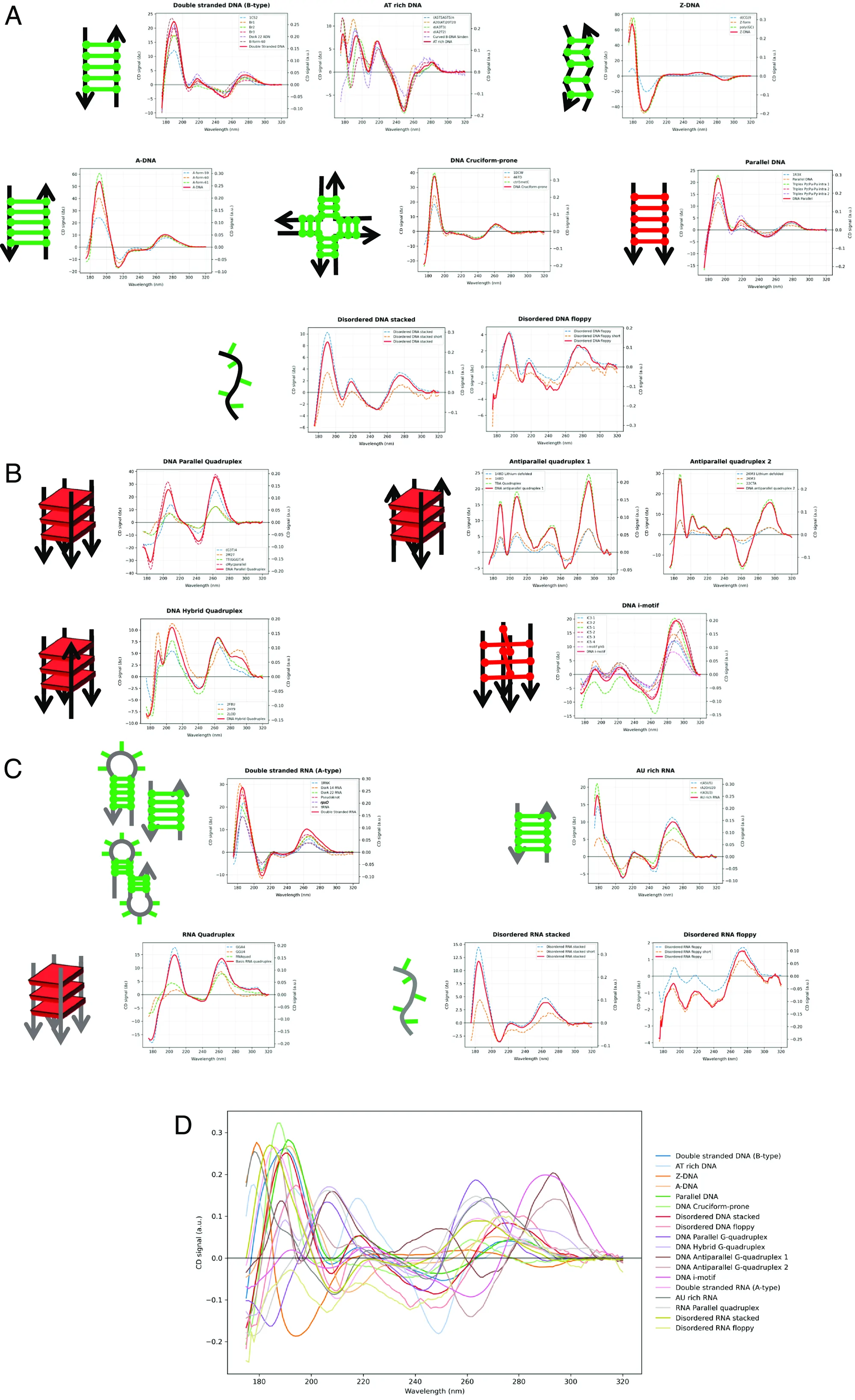
Reference spectra of elementary nucleic acid structures and schematic representations of the corresponding motifs. Panel *A*: DNA; panel *B*: quadruplexes; panel *C*: RNA. DNA and RNA backbones are shown in black and gray, respectively. Green lines indicate Watson–Crick base pairing, red lines denote noncanonical interactions, and red squares represent quadruplexes. In the circular dichroism panels, dashed curves correspond to experimental spectra used to derive the references in Δε units, while the red solid curves show the resulting canonical reference spectra in arbitrary units. It must be noted that RNA generally adopts a conserved A-form helical geometry, resulting in a more limited diversity of CD-resolvable alternative conformations compared with DNA, reflecting intrinsic structural constraints rather than limitations of the approach. For easier comparison of the reference intensities, panel *D* overlays all 18 references on the same graph. This establishes the basis for a comprehensive library of reference spectra for diverse nucleic acid structures, analogous to those available for proteins.

Among the DNA spectral references (1), the canonical B-double-stranded DNA reference displays one strongly positive band at 190 nm, two other positive bands at 218 nm and 275 nm, and one negative band at 252 nm (Fig. 1). In contrast, the Z-DNA reference exhibits a strongly positive band at 180 nm and a positive band at 260 nm, a strongly negative band at 196 nm, and another negative band at 295 nm. The alternative B-DNA reference with AT-rich sequences shows a distinct spectrum with characteristic bands, in particular in the region below 200 nm, which is accessible readily with SRCD. The double-stranded A-DNA also presents a distinctive spectrum, but it forms under low-hydration conditions and in DNA–RNA hybrids (20). While common in these contexts, it is relatively rare for DNA tracts under normal cellular conditions. Nevertheless, certain G-rich tracts, including ones associated with transcription factor IIIA binding, can adopt an A-DNA helix (21–24). The parallel DNA spectrum presents a strongly positive band at 193 nm, two positive bands at 220 and 280 nm, and a negative band at 250 nm. The cruciform-prone DNA reference is characterized by a strongly positive peak at 189 nm, a positive peak at 262 nm, and a negative peak at 239 nm. It is designated as “cruciform-prone” because these sequences tend to adopt cruciform conformations upon crystallization, while also potentially alternating with simple helical forms, possibly in equilibrium (25–27). In particular, for a duplex consisting of stacked B-form helices with localized strand bends at the junction, the CD spectrum would be expected to retain the characteristic features of B-DNA. The pronounced deviations observed in our CD spectra from a canonical B-form signature therefore provides evidence against a purely B-form helical duplex architecture in this system. Importantly, the positions of CD spectral extrema alone are insufficient to uniquely assign nucleic acid conformations. Our previously developed classification algorithm does not rely solely on peak maximum wavelengths, but instead evaluates the complete spectral profile, including relative band intensities and the relationships among positive and negative features across the full wavelength range (15). This approach enables discrimination between conformations with similar peak positions but distinct overall spectral shapes, such as double-stranded helices versus stacked single strands or parallel versus antiparallel B-DNA. Consistent with this, recent studies show that accurate discrimination of G-quadruplex topologies requires analysis of global spectral features rather than single wavelength markers (28).

DNA quadruplexes display considerable structural heterogeneity, with certain spectral signatures being highly sequence-specific (29). For the extant analysis, we restricted analysis to five reference spectra corresponding to the most prevalent DNA quadruplex conformations. The inclusion of additional reference spectra will be considered in future work. The parallel DNA G-quadruplex reference shows two strongly positive peaks at 205 and 265 nm, a strongly negative peak at 185 nm, and another negative peak at 245 nm. The hybrid DNA G-quadruplex spectrum shows two strongly positive bands at 207 and 265 nm, two additional positive bands at 190 and 295 nm, a strongly negative band at 178 nm and one negative band at 240 nm. The first antiparallel DNA G-quadruplex reference exhibits three strong positive peaks at 188, 208, and 294 nm, a lower intensity positive band at 250 nm, and a negative band at 266 nm. The second antiparallel DNA G-quadruplex displays two strong positive peaks at 188 and 294 nm, three additional positive peaks at 202, 217, and 244 nm, a strongly negative peak at 176 nm and a negative peak at 262 nm. Finally, the DNA i-motif reference, a pH-sensitive four-stranded DNA structure formed in cytosine-rich sequences on the complementary strand of G-quadruplexes, displays a strong positive peak at 291 nm, two minor positive peaks at 193 nm and 222 nm, one negative band at 262 nm (possibly composed of two unresolved peaks).

For the RNA reference set, the double-stranded RNA reference presents a strongly positive peak at 187 nm, another positive peak at 265 nm, and a negative band at 210 nm. Notably, we cannot identify a specific signature for A-RNA simple stems, stem-loops (including tRNA), or pseudoknots. The parallel RNA quadruplex reference exhibits positive peaks at 207, 264, and 307 nm, a strongly negative peak at 180 nm and another negative peak at 242 nm. Parallel RNA quadruplexes represent the predominant RNA G-quadruplex family adopting a parallel topology, which is strongly favored by the presence of the 2′-OH group in RNA (30). Similar to DNA AT-rich sequences, the AU-rich reference shows a specific spectrum with two strongly positive peaks at 182 and 266 nm and two negative bands at 208 and 244 nm.

Finally, four random single-stranded NA references were established using randomly synthesized DNA or RNA sequences of 20 and 50 nucleotides (Fig. 1). “Stacked” random references were recorded at 15 °C, a condition that promotes base stacking within the single strand, while “floppy” references were obtained at 95 °C, where base stacking is largely abolished. The stacked random DNA reference shows three positive peaks at 192, 220, and 275 nm, and two negative peaks at 208 and 250 nm. The floppy random DNA spectrum exhibits the same features, slightly shifted toward higher wavelengths and with reduced intensity. The stacked random RNA reference displays a strongly positive peak at 185 nm, another positive peak at 266 nm, and negative peaks at 210 and 240 nm. In contrast, the floppy random RNA reference shows no distinct bands and appears nearly flat, even though subtle features can be discerned in the spectrum (with CD amplitudes close to the noise level of the measurement, typically corresponding to ±1 Δε).

### Development of the Deconvolution Algorithm NA-CDQuant.

Two of the most widely used methods for deriving protein secondary structure from CD spectra are SELCON3 and BeStSel (14, 31). SELCON3 uses a reference dataset of proteins with known structures [SP175 and SMP180, accessible through the PCDDB (13)], selecting spectra similar to the experimental spectrum. It applies SVD to relate the experimental spectrum to the reference set, iteratively adding spectra until valid solutions that are non-negative, sum to approximately 100%, and reconstruct the experimental spectrum within a defined root mean square deviation (RMSD) are obtained. The result averages all valid solutions, reporting proportions of α-helices, β-sheets, turns, and disordered regions. BeStSel also relies on linear deconvolution but with eight predefined reference spectra, optimized for β-sheet subtypes (14). Linear regression against these references reconstructs the CD spectrum and yields secondary structure composition.

Although a refined linear strategy, combining elements of both SELCON3 and BeStSel, improved NA spectra analysis, it did not provide fully satisfactory results. As discussed in Mosca et al. (15), the interpretation of NA CD spectra cannot be considered a purely linear process. We therefore developed a deconvolution method that also includes nonlinear elements to determine the contribution (percentage) of each of the 18 structural references for more complex NA structures (NA-CDQuant algorithm). To test the robustness of NA-CDQuant, we first analyzed NAs whose individual secondary structures had been previously characterized using complementary techniques. Specifically, we examined nine DNA oligonucleotides composed of two major structural elements whose 3D structures were determined by cryotransmission electron microscopy (cryo-TEM) or NMR. The selected synthetic NA structures included one duplex/hybrid quadruplex structure (PDB ID: 8R4E) (32), two duplex/i-motif hybrid structures (PDB ID: 7O5E, 8S4N) (33, 34), two duplex/parallel quadruplex/hybrid quadruplex hybrid structures (PDB ID: 7PNE, 8ABD) (35, 36), one B-DNA/parallel quadruplex hybrid structure (PDB ID: 8DUT) (37), two parallel quadruplexes with floppy tails (PDB ID: 6R9K, 6R9L) (38), and one parallel quadruplex with a B-DNA/stem-loop (PDB ID: 7CLS) (39). For RNA, we used two samples: one polyadenylated hairpin [*rpsO*-polyA, (40)] and RydC small noncoding RNA [PDB ID 4V2S, (41)]. A-DNA is generally found under specialized environmental conditions that are not met during SRCD measurements of the spectra we analyzed. Therefore, the A-DNA reference was excluded from the references used for these specific deconvolutions. It is possible, however, that sequence-dependent tracts of A-DNA may exist within a larger DNA region, but in the present analysis A-tracts have not been included.

**Fig. 2.**
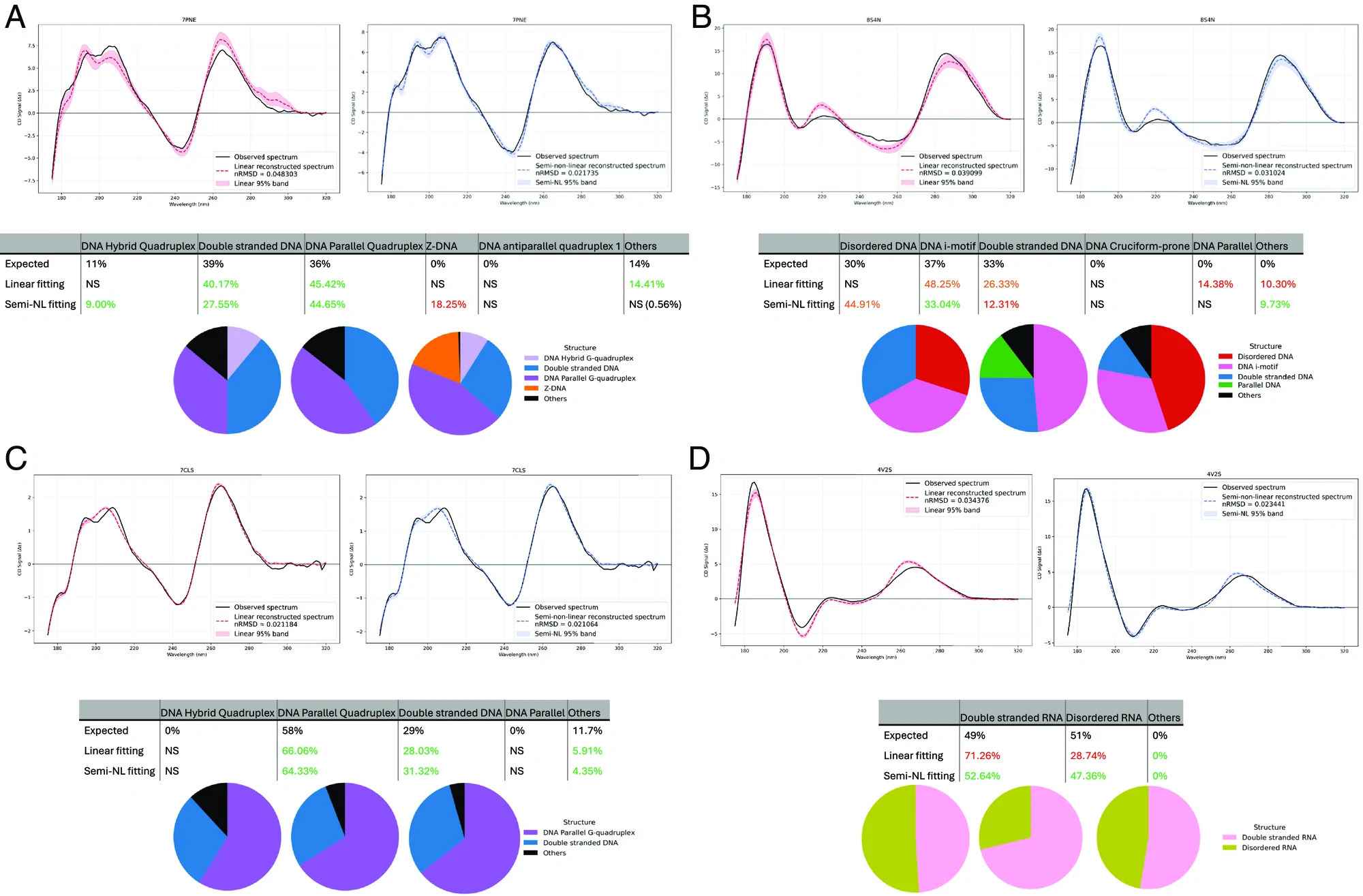
Experimental and fitted circular dichroism spectra for (*A*) duplex/parallel quadruplex/hybrid quadruplex hybrid DNA 7PNE (36 nucleotides), (*B*) a duplex/i-motif hybrid DNA structure 8S4N (43 nucleotides), (*C*) parallel quadruplex with a B-DNA/stem-loop DNA 7CLS (33 nucleotides) and (*D*) the RydC small noncoding RNA 4V2S (65 nucleotides). For each panel, the experimental spectrum is compared with the linear reconstructed spectrum (*Left*) and semi-non-linear reconstructed spectrum (*Right*). Pie charts are also presented below each table (*Left*, theoretical; *Middle*, linear; and *Right*, semi-non-linear). The color code used in pie charts corresponds to that used in Fig. 1*D*. The nRMSD is indicated for each fitting method. The table in each panel reports the proportion of each elementary structure obtained after the deconvolution. Expected results were calculated from the resolved PDB structure by counting the number of nucleotides involved in the substructure. The “others” structure classification corresponds to the proportion that represents less than four nucleotides involved in the substructure. Proportions shown in green are within four nucleotides or within 10% of the expected fit. Proportions shown in orange differ from the expected fit by 4 to 6 nucleotides or 10 to 15%. Proportions shown in red differ by more than six nucleotides or more than 15% from the expected fit. NS, nonsignificant. The number of nucleotides involved is determined by applying the percentage obtained from NA-CDQuant to the total length of the oligonucleotide analyzed. The linear and semi-non-linear 95% bands have been calculated using the bootstrap moving block approach (42).

We first applied the general assumption that the CD spectrum of an NA can be expressed as a linear combination of the 18 reference spectra, similar to the deconvolution approach commonly used for protein CD spectra (14, 31). Importantly, the fitting procedure is constrained by nucleic acid chemistry, using separate DNA and RNA reference libraries to ensure that only structurally compatible conformations are assigned. As illustrated in Fig. 2 and *SI Appendix*, Fig. S1, this initial approach does not fully achieve the desired accuracy, highlighting the limitations of linear decomposition in capturing the complexity of NA conformations. Our complementary semi-non-linear approach, as described below, yields satisfactory results with structural variability, in particular when the temperature at which oligonucleotides structures were determined and the CD spectra acquisition conditions are considered. This method is inspired by linear deconvolution approaches but incorporates nonlinear terms. Thus, the resulting fitted spectrum can be described using the following polynomial expression for linear deconvolution:

[1] $$y_{\mathrm{lin}}=a_{1}x_{1}+a_{2}x_{2}+\cdots +a_{n}x_{n},$$

where the “*a_i_*” values are determined by linear regression and the “*x*” terms correspond to our linear components (the structural references). The nonlinear terms are described as pairwise interaction between references, defined as follows:

[2] $$x_{i,j}={\mathrm{CD}}_{ref,i}*{\mathrm{CD}}_{ref,j}.$$

The inclusion of these interactions terms leads to a new semi-non-linear fitting, which can be expressed as:

[3] $$y_{semi-non-linear}=y_{\mathrm{lin}}+\sum_{i}\;a_{i,i}x_{i,i}+\sum_{i<j}\;a_{i,j}x_{i,j}.$$

To derive the contribution of each structural reference while avoiding double counting of interaction terms, each interaction coefficient *a_i,j_* is equally distributed between the two references involved. The fractional contribution of reference *i* is therefore calculated as:

[4] $$C_{i}=\frac{a_{i}+a_{i,i}+\frac{1}{2}\sum_{j\neq i}^{j}a_{i,j}}{\sum_{k}\left(a_{k}+a_{k,k}\right)+\sum_{k<l}\;a_{k,l}},$$

where the sum of *a_k_* and *a_k,l_* corresponds to the sum of all the coefficients determined in the semi-non-linear fitting. This definition ensures that each coefficient is counted only once in the total composition and that the sum of the fractional contribution of all structural references is equal to 1. The percentage contribution of each reference is obtained by multiplying *C_i_* by 100.

Components are then selected using a forward stepwise selection method, evaluating different models by progressively adding the best component at each iteration. This method is a sequential, data-driven variable selection procedure commonly used in statistics and machine learning, particularly for linear regression models to identify parsimonious subsets of predictors (43), here our reference spectra. The selected references are those which best describe the variation observed in our spectra. The reference selection is stopped when an information criterion becomes constant. This information criterion in our selection is the Bayesian Information Criterion (BIC) (44). The BIC reflects the trade-off between the error of the model, related to the RMSD, and a penalty related to the model complexity. The composition is then obtained by adding the a_i_ values of the linear reference together with the a_i,j_ values of every nonlinear term involving that reference. The percentage is calculated by taking the ratio of this sum by the total sum of all the “a” coefficients. All percentages result from bootstrap resampling using a moving block approach, selected among several tested strategies for providing the highest precision, defined as the percentage of experimental spectrum points within the CI. This approach ensures that our results do not depend on a single estimation across the whole spectrum, that our coefficients remain robust, and that the risk of biased values arising from the analysis of a single sample is avoided. This method achieves significantly improved normalized RMSD (nRMSD) values compared to conventional techniques, thereby extending accuracy and reliability in CD-based structure prediction.

As shown in Fig. 2, the nRMSD decreases from 0.048 to 0.022 for a duplex/parallel quadruplex/hybrid quadruplex hybrid structure 7PNE, from 0.039 to 0.031 for a duplex/i-motif hybrid structure 8S4N, from 0.031 to 0.023 for the RydC small noncoding RNA 4V2S. For the parallel quadruplex with B-DNA/stem-loop 7CLS, the nRMSD shows no major improvement as the linear fitting already succeeded in evaluating the composition. The deconvolution results for the other spectra used to test the robustness of the method are shown in the *SI Appendix*, Fig. S1. The expected compositions present in each table included in Fig. 2 and *SI Appendix*, Fig. S1 were calculated by counting the number of nucleotides involved in each elementary structure present in the resolved model available on the PDB. A Pearson correlation coefficient was also calculated between the predicted and expected secondary-structure fractions across the complete validation set (*SI Appendix*, Table S2). While the nRMSD measures the agreement between the experimental and reconstructed CD spectra, the Pearson correlation coefficient evaluates the relationship between the predicted and expected secondary-structure fractions.

It should be noted that the extended far-UV range provided by SRCD improves the accuracy of NA-CDQuant deconvolution. Analysis of spectra truncated to the wavelength range typically accessible with conventional CD instruments revealed reduced fitting accuracy and less reliable conformational population estimates, highlighting the added value of SRCD data (*SI Appendix*, Table S2). Nevertheless, NA-CDQuant remains applicable to conventional CD spectra and provides meaningful conformational estimates, although with reduced accuracy compared with SRCD-based analyses.

NA-CDQuant additionally incorporates optional physicochemical and sequence-based selection criteria to ensure that only structurally plausible DNA conformations are considered during analysis. Specifically, the Z-DNA component is included for sequences containing (GC)_n_, (CG)_n_, (GT)_n_, or (TG)_n_ repeats, which have the potential to adopt the Z-DNA conformation (45), while allowing its exclusion when such sequence features are absent. Similarly, the A-DNA component is considered primarily under dehydrating conditions or other specific environmental conditions that favor A-form DNA (21–24). In addition, sequence-based criteria are proposed to restrict the prediction of G-quadruplex and i-motif contributions to sequences containing guanine or cytosine tracts (with at least G2 or C2, respectively), thereby improving the biological relevance of the conformational assignments. Note that applying the sequence-based criterion to spectra with a non-zero-fitted Z-DNA contribution (7PNE, Fig. 2 or 8DUT, *SI Appendix*, Fig. S1) showed that omitting the Z-DNA component substantially worsened the fit. Given the sequence context and the relatively small fitted Z-DNA fractions, we do not interpret these results as evidence for a fully formed Z-DNA structure. No sequence element in 7PNE has the properties required to form Z-DNA. 8DUT does contain a potential Z-DNA forming tract, but formation is unlikely in conditions used for CD measurement. Rather, we suggest that in these complex structures the spectra may reflect local helical or other structural disruptions that give rise to a “Z-like” circular dichroism signature.

**Fig. 3.**
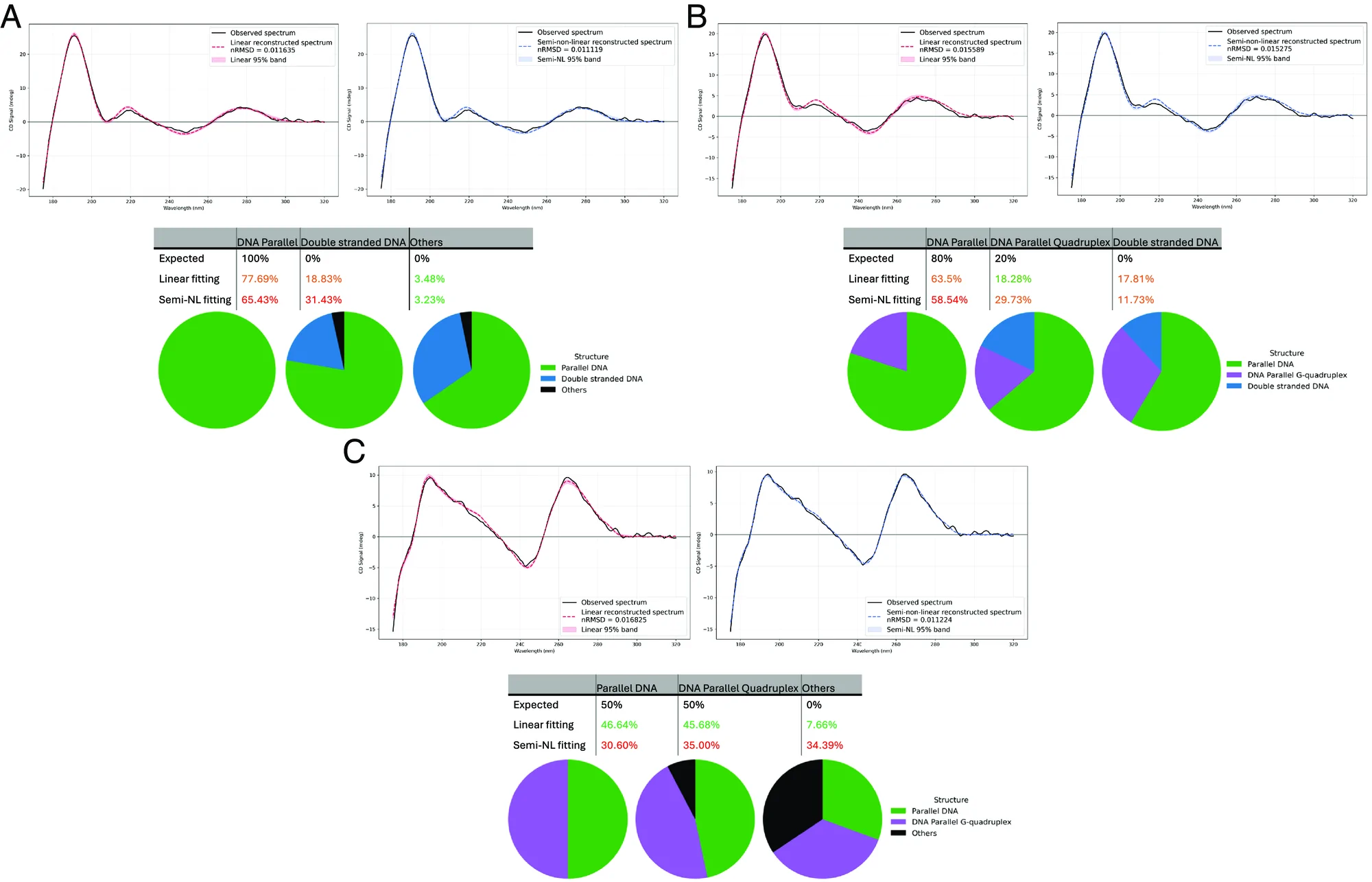
Quantitative deconvolution of coexisting nucleic acid conformations by NA-CDQuant. Defined mixtures of reference structures, a parallel DNA (GAAGAAGAAGAAG+CTTCTTCTTCTTC) and quadruplex DNA [parallel, TT(GGGT)_4_] were prepared at ratio (*A*) 100/0%, (*B*) 80/20%, and (*C*) 50/50% and analyzed by SRCD spectroscopy. Pie charts are also presented below each table (*Left*, theoretical; *Middle*, linear; *Right*, semi-non-linear). The color code used in pie charts corresponds to that used in Fig. 1*D*. Here, we observe that when two independent sequences are mixed in solution, intramolecular interactions between them are absent, and the linear model provides a more accurate description of the resulting data.

Additionally, we evaluated the ability of NA-CDQuant to resolve coexisting NA conformations in solution. To this end, we prepared defined mixtures of two reference structures, namely, a DNA with a parallel orientation and a parallel DNA quadruplex, at varying proportions (100/0%, 80/20%, and 50/50% parallel DNA/quadruplex ratio, respectively) and analyzed their composite SRCD spectra. As shown in Fig. 3, NA-CDQuant accurately recovered the relative contributions of each conformation across all tested ratios using a linear fitting (Eq. **1**, with *a_I_* as constants), demonstrating its robustness and quantitative reliability. These results confirm that the algorithm can determine the fractional populations of coexisting structures within a mixture. This capability is particularly important for nucleic acids, which frequently populate dynamic conformational ensembles in solution rather than a single static state (46).

The choice of the spectral decomposition approach is thus closely linked to the structural organization and physical properties of the system under investigation. The differing performance of the linear and semi-non-linear approaches primarily reflects these underlying characteristics. The linear model is best suited for mixtures of structurally independent species, where the measured CD spectrum can be accurately represented as the sum of individual component spectra (Fig. 3). In contrast, the semi-non-linear approach is better suited for systems containing multiple covalently linked structural elements, where intramolecular coupling can modify local CD responses and lead to deviations from simple spectral additivity (Fig. 2 and *SI Appendix*, Fig. S1).

## Discussion

In this study, we establish a systematic framework for quantitative interpretation of NA CD spectra by defining 18 structurally representative SRCD reference spectra. A large dataset is required for NAs due to their immense conformational diversity. While protein CD signals in the 170 to 260 nm region largely arise from the repetitive ~2 Å peptide bond, NA chromophores comprise phosphate, sugar, and base with over 9 Å for each nucleotide, absorbing between 320 to 170 nm. Because ab initio prediction of CD spectra remains out of reach for most proteins and NAs (47), experimentally standardized spectra with well resolved structures currently provide the most reliable foundation for NA folding assessment. The reference spectra presented here span the major DNA and RNA conformations with experimentally controlled sequences representing extremes of base stacking behavior. These include canonical helices, G-quadruplexes, i-motifs, cruciform-prone, AU- and AT-rich sequences, and RNA motifs such as parallel quadruplexes and stem-loops. By capturing this breadth of structural diversity, the reference library (*SI Appendix*, Table S1) provides a standardized spectral vocabulary for NAs, analogous to the SP175/SMP180 and PCDDB reference sets used in protein CD analysis, and establishes a foundation for robust, reproducible spectral interpretation.

Our algorithm, NA-CDQuant, extends the linear decomposition strategies traditionally used for proteins, such as SELCON3 and BeStSel, by incorporating nonlinear interaction terms and a stepwise Bayesian Information Criterion-based selection of components. While linear regression alone can approximate NA secondary structure, the complex spectral overlap and cooperative interactions inherent to NAs often result in incomplete or inaccurate quantification. NA-CDQuant overcomes these limitations, producing improved nRMSD values across diverse DNA and RNA structures, including hybrid, multistranded, and partially folded species. This allows accurate determination of fractional contributions of multiple structural motifs within a composite CD spectrum, even under near-physiological conditions. Importantly, nucleic acid CD spectra are generally considered to primarily reflect helical structure and conformational features rather than sequence contribution alone. This was demonstrated by three duplex oligonucleotides with identical base composition, (GCGCTAGCGC), (CCCCTAGGGG), and (GGGGTACCCC), which exhibited distinct CD spectra corresponding to B-form, A-form, and intermediate conformations (22). Similarly, (GC)n repeats can adopt either B-DNA or Z-DNA depending on ionic conditions (45), and G-quadruplexes can transition between different topologies with distinct CD signatures (48). Thus, CD spectra primarily reveal nucleic acid structural conformation, with sequence influencing spectra through its effects on conformational preferences.

NA-CDQuant accurately described structures in many model NAs tested. For complex structures however, the ability of NA-CDQuant to achieve precise agreement between actual and predicted structural assignments, including the presence or absence of specific conformations, may be limited. For example, Z-DNA was predicted, as discussed above, in two complex structures (7PNE and 8DUT) in which it is not expected. In other complex sequences (8R4E and 7O5E), dsDNA and disordered DNA were under- and overrepresented, respectively. Circular dichroism (CD) spectra represent ensemble-averaged measurements of nucleic acids in solution and therefore inherently reflect conformational dynamics. Structural fluctuations, perturbations of local structures arising from flanking-sequence dynamics, and molecular interactions may contribute to unique CD spectral features in complex structures. In particular, challenges associated with describing structural interfaces and transitions between distinct structural states, as well as limitations inherent to the NMR fitting procedure (49), may contribute to the observed discrepancies for 8R4E and 7O5E. Consequently, these differences should be interpreted with caution, as perfect agreement between predicted and expected structural fractions may not necessarily be expected for structures containing multiple consecutive structural motifs. Ultimately, a better understanding of the effects of dynamic structural transitions on these complex experimental structures may improve the accuracy of the analysis.

By enabling quantitative deconvolution of complex spectra, NA-CDQuant captures the dynamic structural ensembles that NAs populate in solution. Unlike static crystallographic snapshots, CD spectroscopy reports how these molecules respond to environmental changes such as ionic strength, temperature, or ligand binding, making it uniquely suited to study functional structural dynamics. This capability is particularly relevant for NA biology. The ability to detect subtle conformational shifts, including noncanonical quadruplexes or partially folded intermediates, addresses a critical need for quality control and rational design in pharmaceutical development. A key challenge for future work will be resolving the spectra of NA that contain combinations of five or more structural motifs. Nevertheless, the integration of deep learning approaches with additional predictive models should enable accurate deconvolution and analysis of such complex spectral ensembles in the future.

The results of this study therefore open several promising avenues for future development. Integration with complementary spectroscopic and computational tools, including FTIR spectroscopy and molecular dynamics simulations, should offer the potential to achieve a complete view of NA folding and stability. Expanding the reference library to include additional complex and hybrid structures will increase the algorithm’s capacity to classify subtle structural variations. Incorporating posttranscriptional and epigenetic modifications, such as pseudouridylation and methylation, will further enhance the relevance of the method to cellular and therapeutic RNAs. Finally, the development of an open-access computational platform embedding NA-CDQuant and the reference spectra database will make this approach broadly accessible to the scientific community, enabling rapid, reliable, and quantitative CD-based analysis across both fundamental research and therapeutic applications.

In summary, the combination of a curated SRCD reference library with the NA-CDQuant algorithm transforms CD spectroscopy into a predictive, quantitative tool for NAs. It bridges a longstanding gap between qualitative spectral interpretation and rigorous structural analysis, opening avenues for studying NA dynamics, guiding therapeutic design, and supporting regulatory and quality control frameworks in the emerging field of NA–based medicines.

## Materials and Methods

### Sample Preparation.

The analyzed samples consisted mainly of synthetic oligonucleotides (*SI Appendix*, Table S1) (Eurogentec, Belgium). Unless otherwise specified, they were resuspended in nuclease-free Molecular Grade Water nuclease-free water (Sigma Aldrich) at 1 mM (expressed in strand concentration, based on OD measurements and oligonucleotides molar extinction coefficient). The oligonucleotides were analyzed after annealing and relaxation by heating at 95 °C for 2 min followed by gradual cooling to 25 °C at an approximative rate of 1 °C per minute. When relevant, duplex formation was confirmed via native PAGE analysis (50). For quadruplex–forming sequences requiring specific ionic conditions, oligonucleotides were resuspended in 10 mM potassium phosphate buffer (pH 7.4) with appropriate salts. Before SRCD analysis, G-quadruplex formation was confirmed *via* native PAGE analysis (51).

### SRCD Analysis.

SRCD measurements were performed at the DISCO beamline at Synchrotron SOLEIL, France and at the AU-CD beamline on ASTRID2, Aarhus University, Denmark, following the procedure outlined in (52, 53). For Synchrotron SOLEIL experiments, approximately 4 µL of sample was placed into a CaF_2_ circular cell (ranging from 1 to 50 µm pathlengths) (54). Spectra were acquired in triplicate at 1 nm intervals with an integration time of 1.2 s, covering the range from 170 to 320 nm. Calibration of amplitude and wavelength positions was carried out using (+)-camphor-10-sulfonic acid (CSA). Data analysis, including averaging, baseline subtraction, smoothing, scaling, and spectral summation, was performed using CDtoolX (55). CD spectra acquired on ASTRID2 were measured in the range 165 to 320 nm using the fast-scanning mode, whereby the monochromator is continuously moving and the data binned into 1 nm steps. Six scans each of the sample and an appropriate reference baseline were measured at a speed of 142 nm/min. They were then averaged and subtracted to give the final spectrum.

### Model Definition.

NA-CDQuant forward stepwise selection has been performed as follows. First, we made the assumption that our spectra are well centered at 0 Δε at wavelengths between 317 nm and 320 nm, which leads to an initialization needing no intercept. Then, our first step is to evaluate the first candidate reference spectrum as the closest reference to the spectrum we are deconvolving. Here, we calculate the first factor for our predictive model as a one-factor model. Once the first model is made, we calculate the performance of our model using nRMSD and BIC. nRMSD estimates the deviation between the original experimental spectrum and the fitted one and is calculated as

[5] $$\mathrm{nRMSD}=\frac{\mathrm{RMSD}}{max\;CD_{\exp}-min\;CD_{\exp}},$$

where RMSD is:

[6] $$\mathrm{RMSD}=\sqrt{\frac{1}{n}\sum_{i=1}^{n}{\left({\mathrm{CD}}_{exp,i}-CD_{fit,i}\right)}^{2}}.$$

The BIC works by combining two elements: the complexity penalty (CP) of the model, expressed as:

[7] $$C_{p}=k\;\text{log}(n),$$

and the residual sum of squares (RSS) which reflects the error of the model:

[8] $$\mathrm{RSS}=\sum_{i=1}^{n}{\left({\mathrm{CD}}_{exp,i}-{\mathrm{CD}}_{fit_{i}}\right)}^{2}.$$

Therefore, it decreases when the model error gets smaller and increases when the model becomes too complex by adding more reference spectra. As a result, the best model is the one with the lowest BIC:

[9] $$\mathrm{BIC}=n\;\text{log}\left(\frac{\mathrm{RSS}}{n}\right)+C_{p},$$

meaning it fits the model well without becoming unnecessarily complicated. In the formula, *n* is the wavelength range of the spectrum and *k* is the number of parameters used to fit the spectrum.

When our first model is defined, NA-CDQuant determines the final fitting model by progressively adding reference spectra one by one, from the closest to our experimental spectrum until BIC becomes constant.

### Statistical Analysis.

Confidence in the final model is determined by the bootstrap method (42). Bootstrap is a resampling-based statistical method used to assess the stability, variability, and predictive performance of a regression model. It relies on repeatedly generating new datasets by sampling with replacement from the original data, allowing estimation of model uncertainty without requiring additional experimental measurements.

The bootstrap is performed following several steps; first step is the resampling of our dataset. We generate an optimized set of subset spectra from our experimental spectra, determined taking its wavelength window into consideration. The wavelength window of the subset spectra is also optimized in order to avoid oversampling. In our case, we randomly generated 20 subset spectra with a random wavelength window of 64 nm by using a mobile block approach. We then have 20 subset spectra that are fitted using the model previously selected using the whole experimental spectrum.

Second step, for each subset of spectra the parameters *a_i_* of the model are then recomputed resulting in 20 estimations of our parameters. Finally, we determine a SE over the B = 20 estimated parameters as follows:

[10] $$\mathrm{SE}\left(a_{i}\right)=\sqrt{\frac{1}{B-1}\sum_{i=1}^{B}{\left(a_{i}-\bar{a_{i}}\right)}^{2}}.$$

The final CI is defined as

[11] $$\bar{a_{i}}\pm z_{1-\alpha /2}\;\mathrm{SE}\left(a_{i}\right).$$

In our case, α is equal to 5% therefore $z_{1-\alpha /2}$ is equal to 1.96. The wavelength window size, the number of subset spectra, and the α value can be modified by the user.

## Conclusion

In conclusion, the combination of SRCD reference spectra with the NA-CDQuant algorithm establishes a robust and quantitative framework for resolving complex or coexisting nucleic acid (NA) conformations in solution. By capturing structural ensembles rather than single averaged states, this approach provides detailed insight into conformational equilibria, folding pathways, and stability landscapes, as well as into molecular recognition processes. Such information is directly relevant to the rational design and optimization of DNA- and RNA-based therapeutics. Moreover, the method’s rapid, nondestructive, and predictive nature makes it well suited for routine analytical workflows. It enables efficient formulation screening, batch-to-batch quality control, and mechanistic investigations under physiologically relevant conditions. Together, these features offer a practical and scalable strategy to accelerate the development, characterization, and regulatory advancement of nucleic acid–based medicines.

## Supplementary Material

Appendix 01 (PDF)

## Data Availability

Python implemented script of the workflow can be downloaded from https://github.com/Sanofi-Public/CD-spectra-deconvolution (56). License conditions apply, including limitation to noncommercial uses only.
