# Supplementary material for: NA-CDQuant: Quantitative spectral analysis of nucleic acid structure by circular dichroism spectroscopy
Source: Proc Natl Acad Sci U S A. 2026 Oct 6;123(41):e2617882123. doi: 10.1073/pnas.2617882123 (PMC13663790; doi:10.1073/pnas.2617882123)
Supplement: Supplementary file 1 [file pnas.2617882123.sapp.pdf]

PNAS

Supporting Information for

NA-CDQuant: Quantitative Spectral Analysis of Nucleic Acid Structure by Circular Dichroism Spectroscopy

Kevin Mosca, Søren Vrønning Hoffmann, Nykola C. Jones, Richard R. Sinden, Frank Wien, Sergio Marco and Véronique Arluison

Corresponding author: Veronique.arluison@cea.fr

This PDF file includes:

Supplementary Figure S1

Supplementary Tables 1 and 2

Supplementary Figure S1

Supplementary Figure S1. Experimental and fitted circular dichroism spectra for (A, B) parallel quadruplexes with floppy tails (6R9K (25 nucleotides), 6R9L (25 nucleotides)), (C) a duplex/i-motif hybrid structure (7O5E (25 nucleotides)), (D) a duplex/parallel quadruplex/hybrid quadruplex hybrid structure (8ABD (36 nucleotides)), (E) a duplex/hybrid quadruplex structure (8R4E (27 nucleotides)), (F) a B-DNA/parallel quadruplex hybrid structure (8DUT (46 nucleotides)), and (G) a polyadenylated hairpin (*rpsO-polyA* (50 nucleotides)). For each panel, the experimental spectrum is compared with the linear reconstructed spectrum (left) and semi-non-linear reconstructed spectrum (right). The nRMSD value is indicated for each fitting method. The table in each panel reports the proportion of each elementary structure obtained after the deconvolution. Expected results were calculated from the resolved PDB structure by counting the number of nucleotides involved in the substructure. The “others” structure classification corresponds to the proportion that represents less than 4 nucleotides involved in the substructure. Proportions shown in green are within 4 nucleotides or within 10% of the expected fit. Proportions shown in orange differ from the expected fit by 4–6 nucleotides or 10–15%. Proportions shown in red differ by more than 6 nucleotides or more than 15% from the expected fit. NS, non-significant. The linear and semi semi-non-linear 95% bands have been calculated using the bootstrap moving block approach.

A

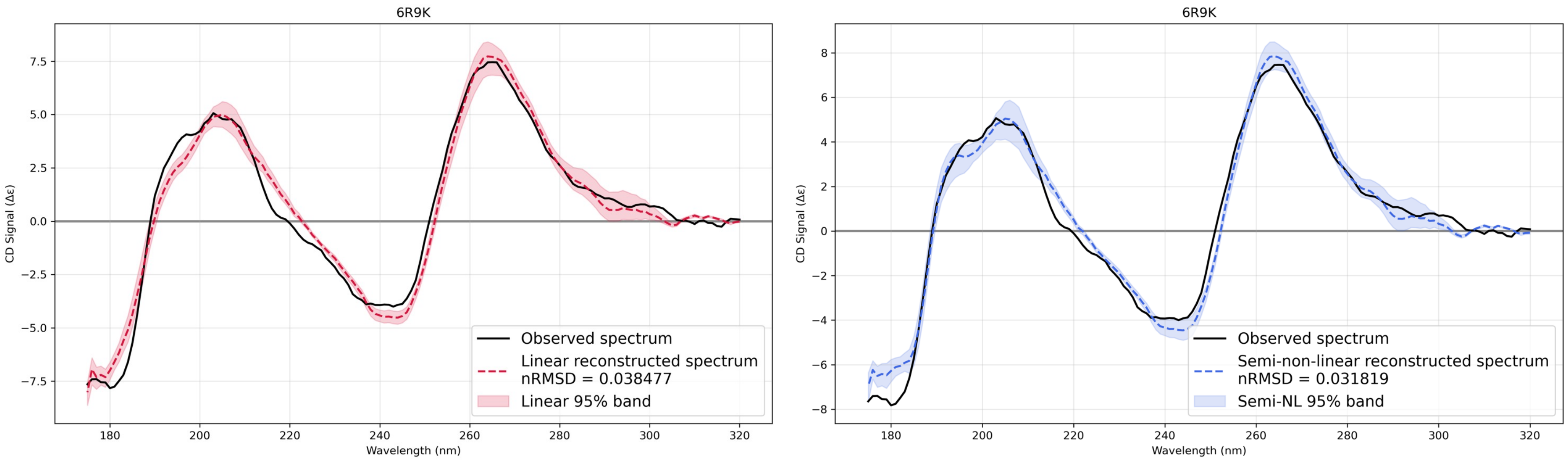

	DNA Parallel Quadruplex	Disordered DNA	DNA Cruciform-prone	DNA antiparallel quadruplex 1	Others
Expected	68%	32%	0%	0%	0%
Linear fitting	64.65%	21.96%	NS	NS	13.29%
Semi-NL fitting	72.89%	14.83%	NS	NS	9.22%

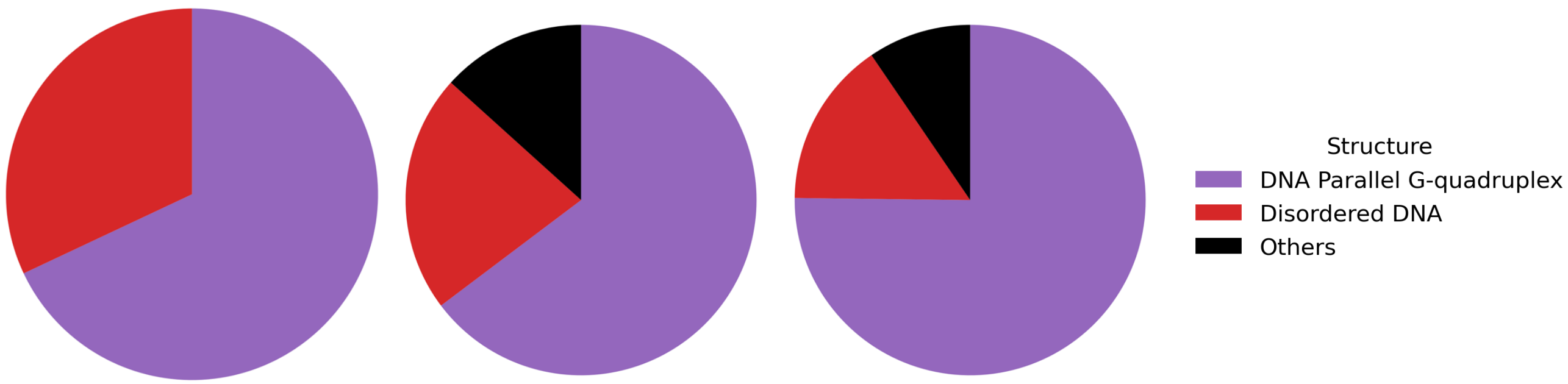

B

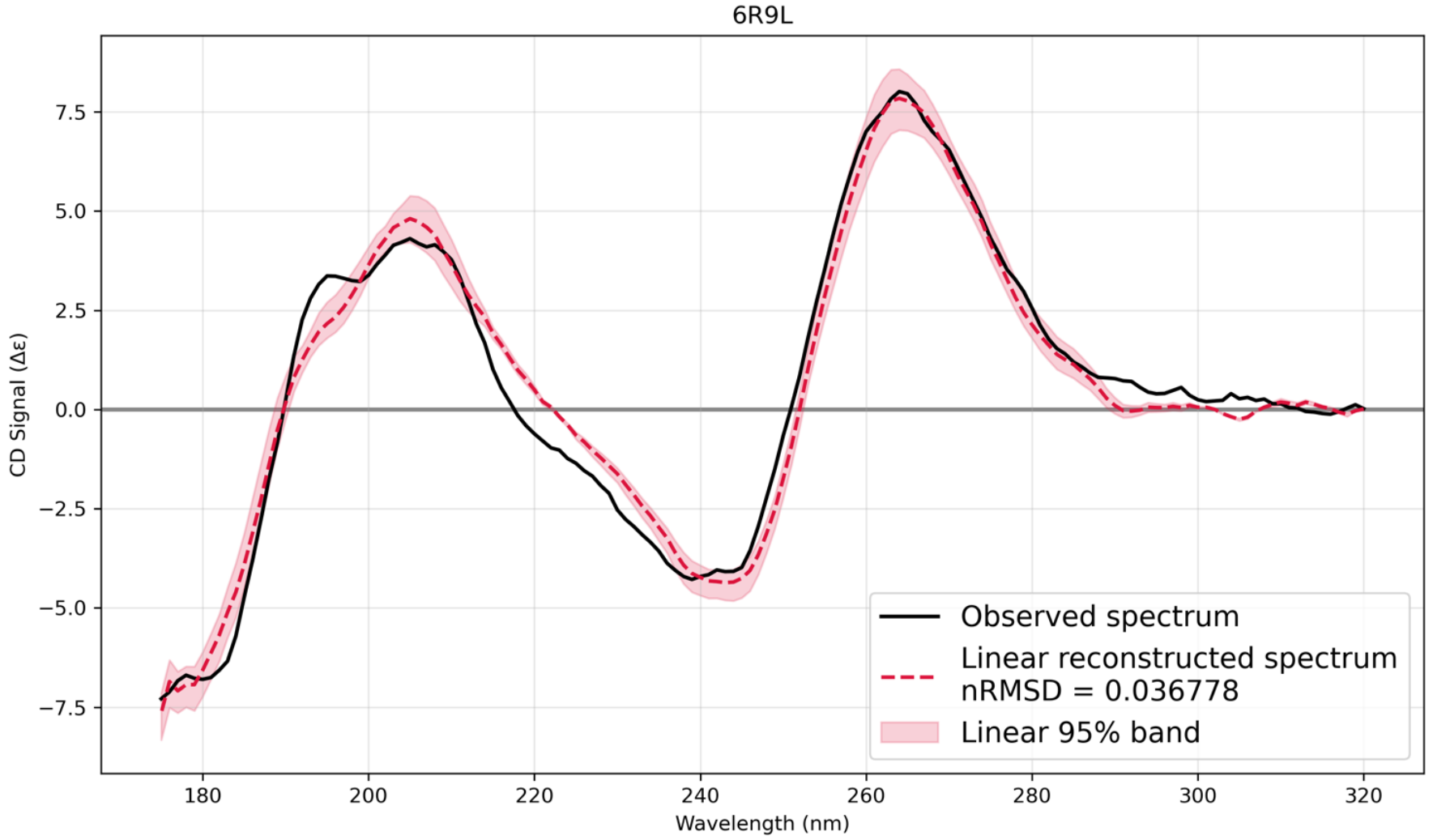

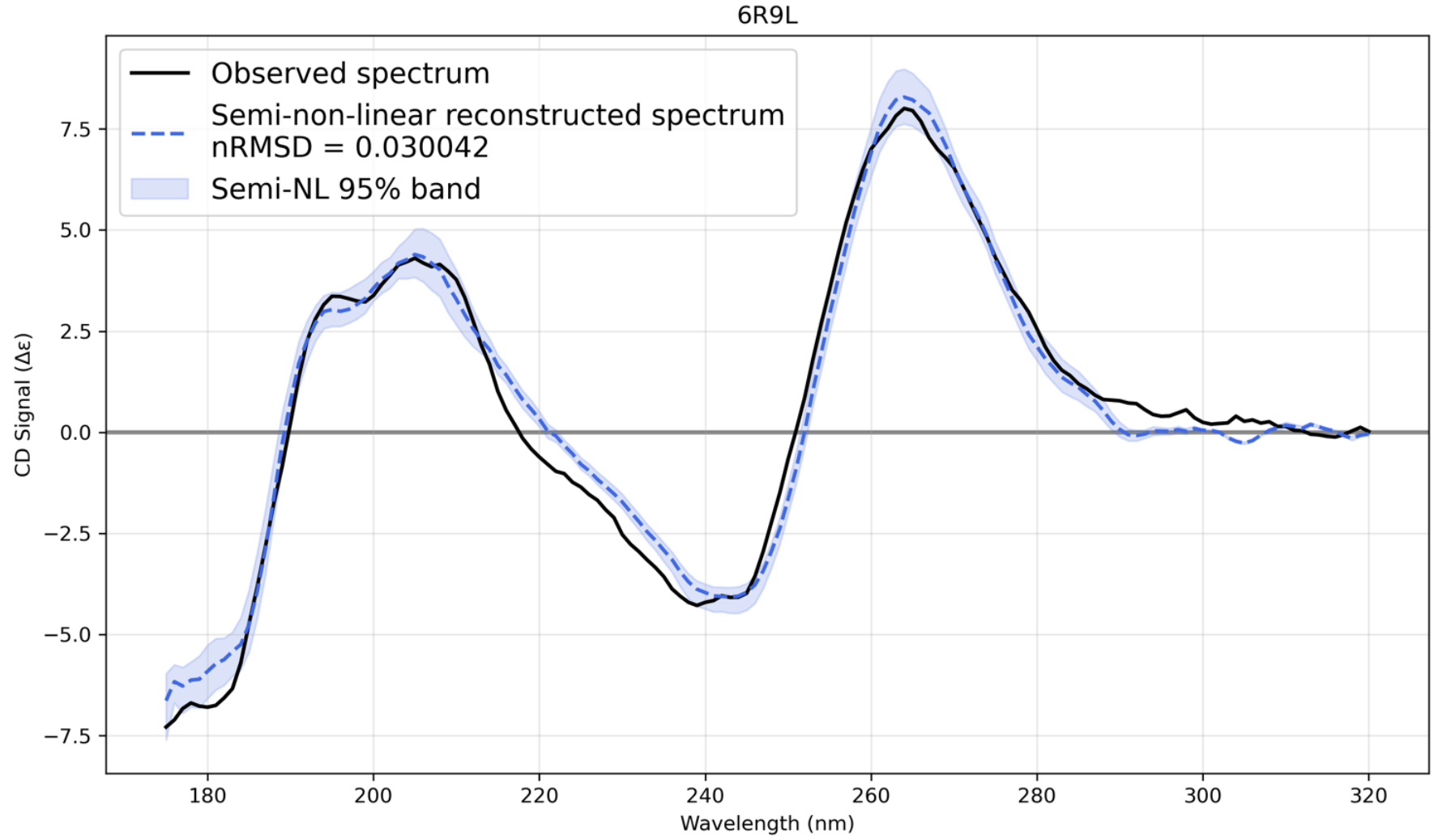

	DNA Parallel Quadruplex	Disordered DNA	DNA Cruciform-prone	Others
Expected	71%	29%	0%	0%
Linear fitting	68.83%	18.41%	12.76%	0%
Semi-NL fitting	75.30%	14.50%	10.21%	0%

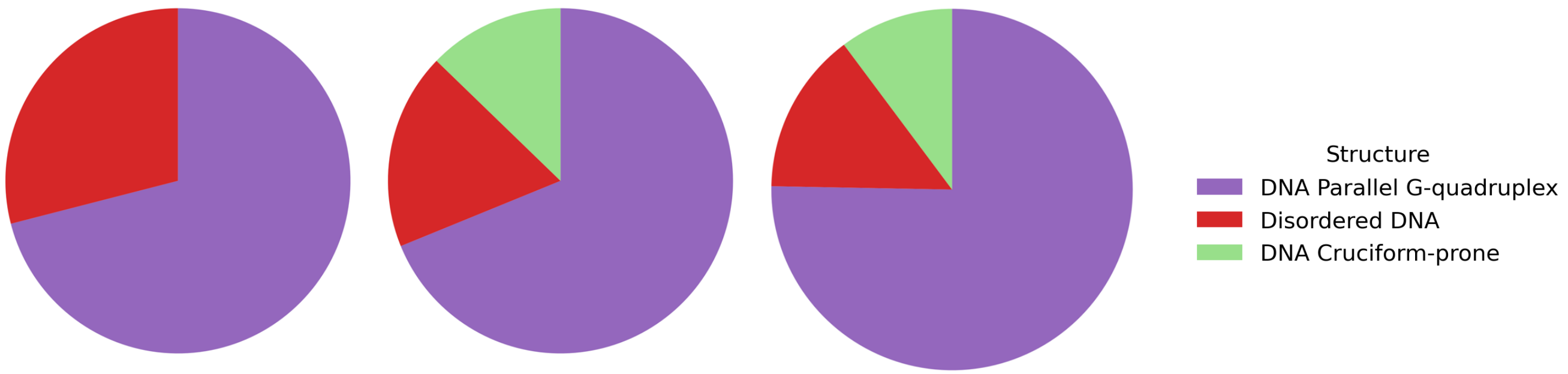

C

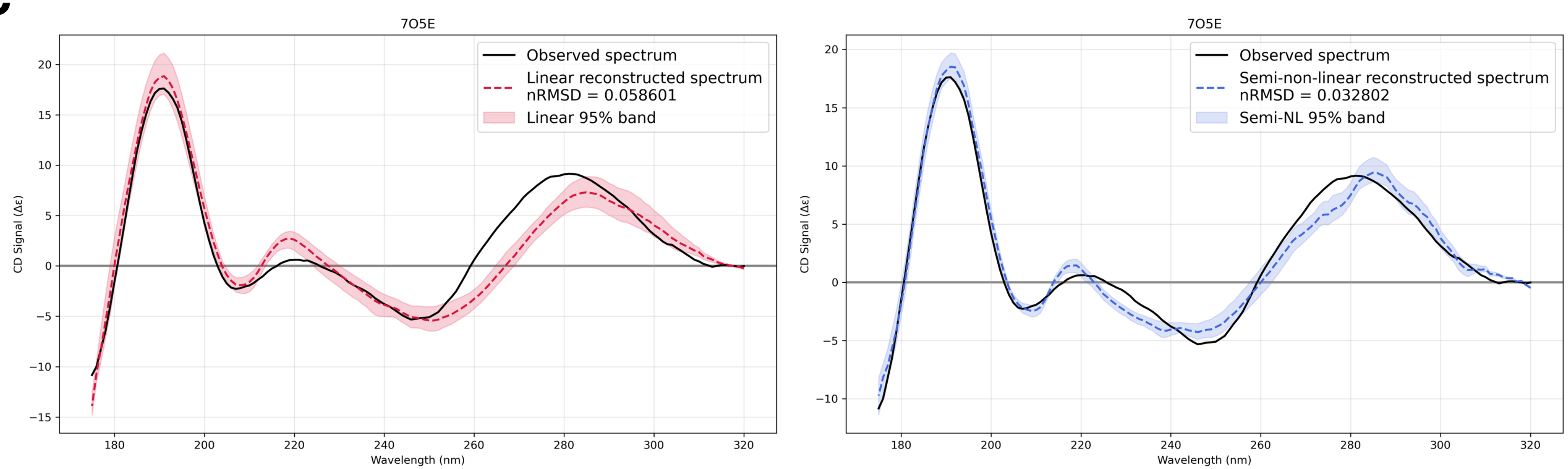

	Disordered DNA	DNA i-motif	DNA Cruciform-prone	Double stranded DNA	DNA Parallel	Others
Expected	23%	33%	0%	44%	0%	0%
Linear fitting	28.19%	20.14%	15.29%	28.14%	NS	0%
Semi-NL fitting	46.26%	39.87%	NS	NS	NS	13.87%

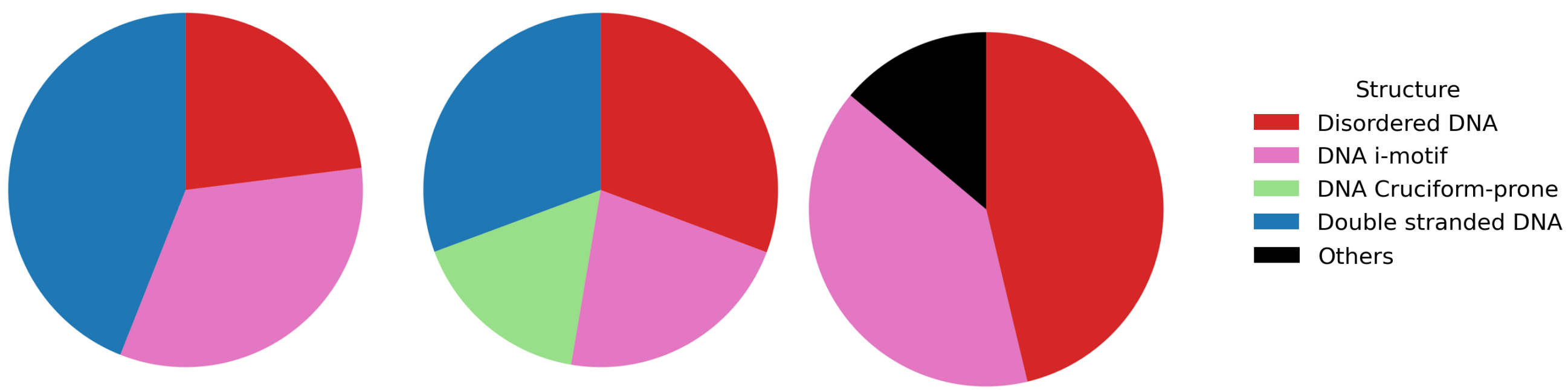

D

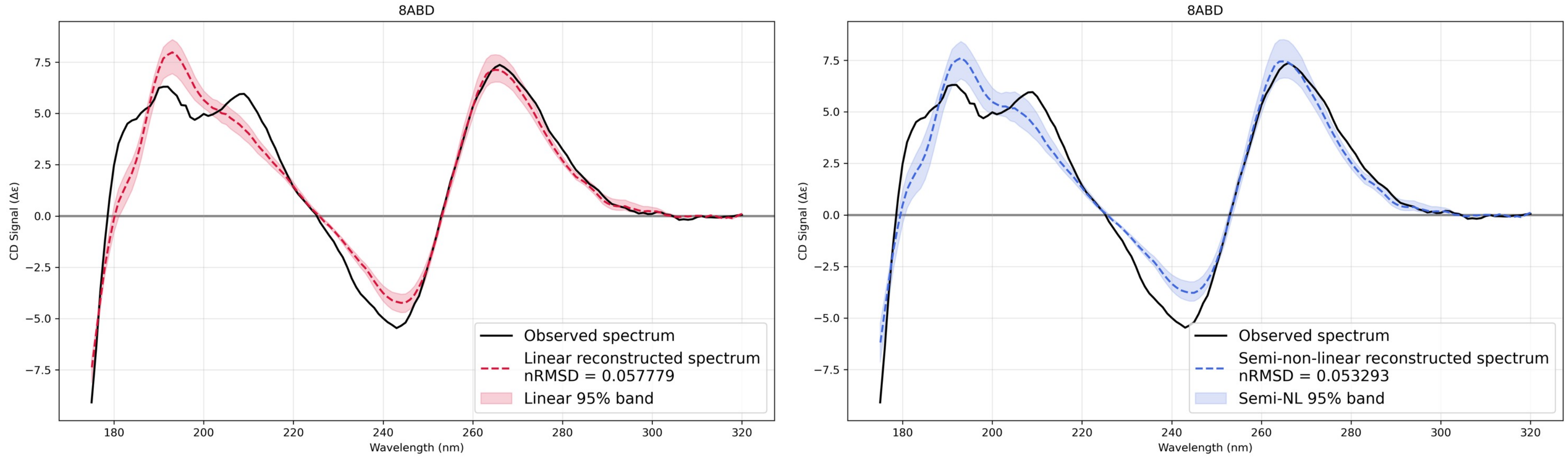

	DNA Hybrid Quadruplex	Double stranded DNA	DNA Parallel Quadruplex	Others
Expected	11%	47%	36%	6%
Linear fitting	NS	50.25%	46.76%	2.99%
Semi-NL fitting	15.87%	41.57%	26.71%	0%

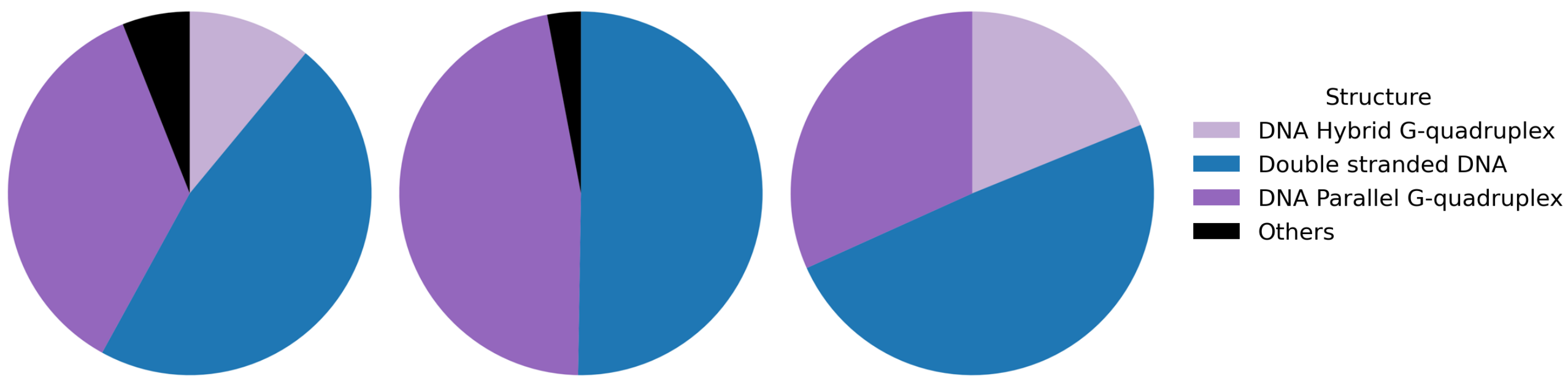

E

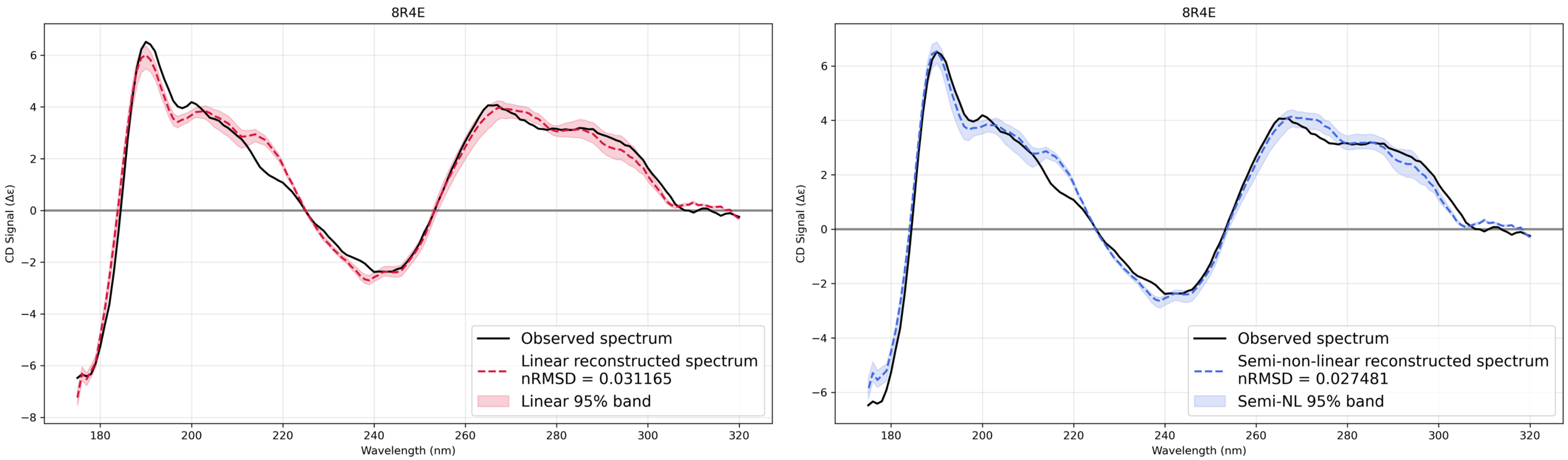

	DNA Hybrid Quadruplex	Double stranded DNA	Disordered DNA	DNA Cruciform-prone	DNA antiparallel quadruplex 1	Others
Expected	44%	30%	0%	0%	0%	25.56%
Linear fitting	49.40%	NS	31.52%	NS	NS	19.08%
Semi-NL fitting	50.59%	NS	24.69%	NS	18.60%	6.12%

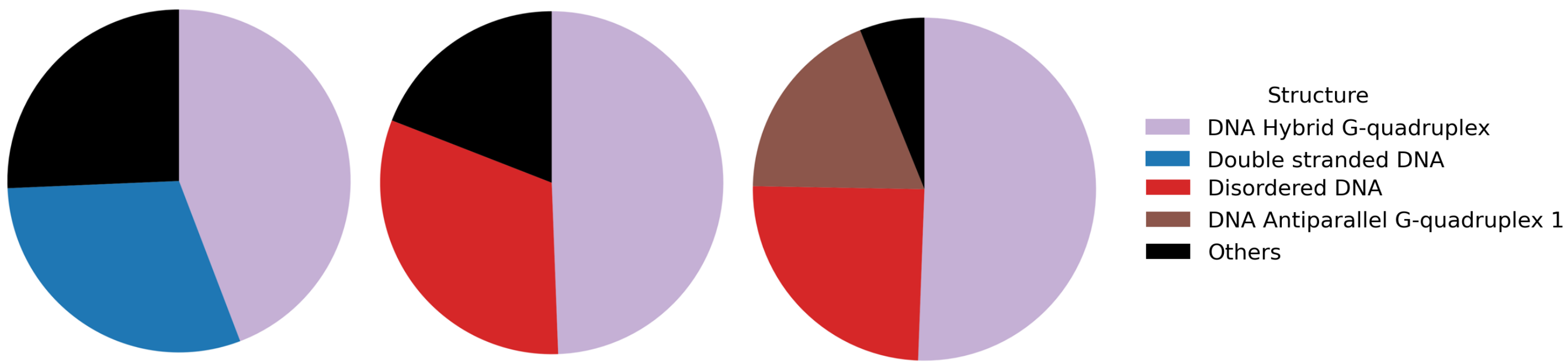

F

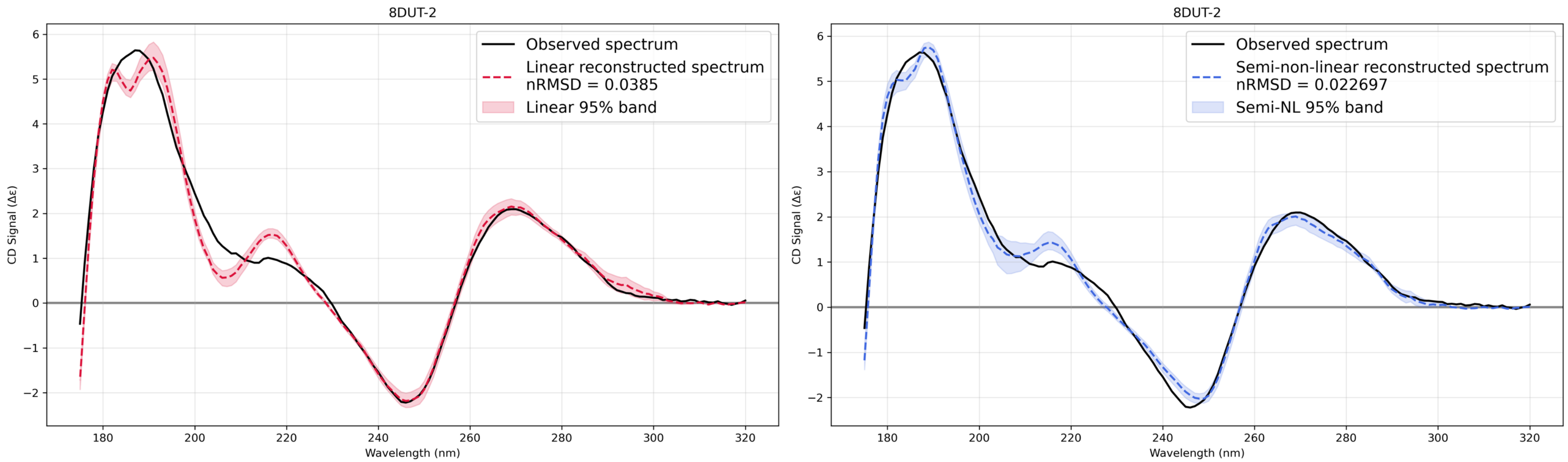

	Double stranded DNA	DNA Parallel Quadruplex	Z-DNA	AT rich DNA	DNA antiparallel quadruplex 1	Others
Expected	59%	17%	0%	11%	0%	13%
Linear fitting	54.15%	18.46%	15.55%	7.87%	NS	3.95%
Semi-NL fitting	45.20%	24.74%	29.20%	4.45%	NS	0.86%

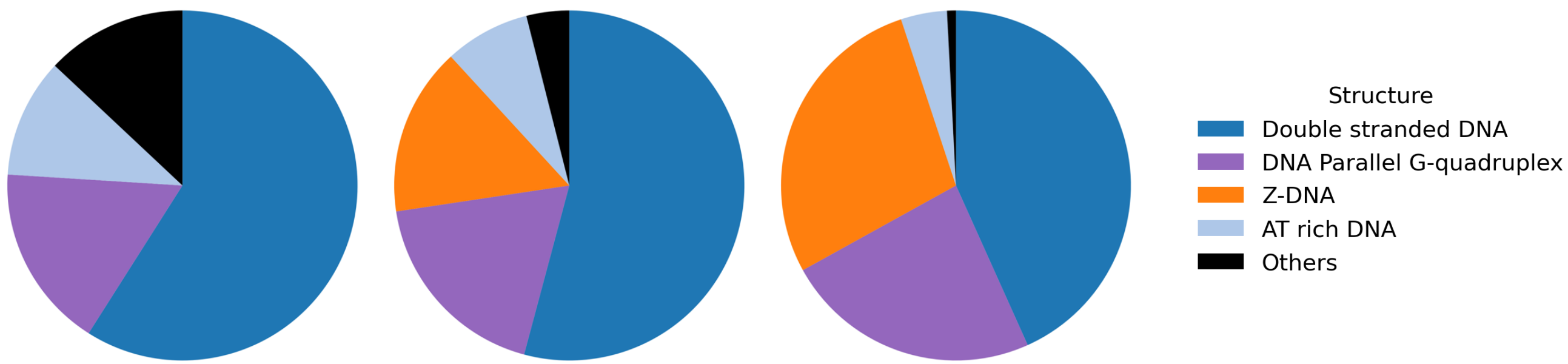

G

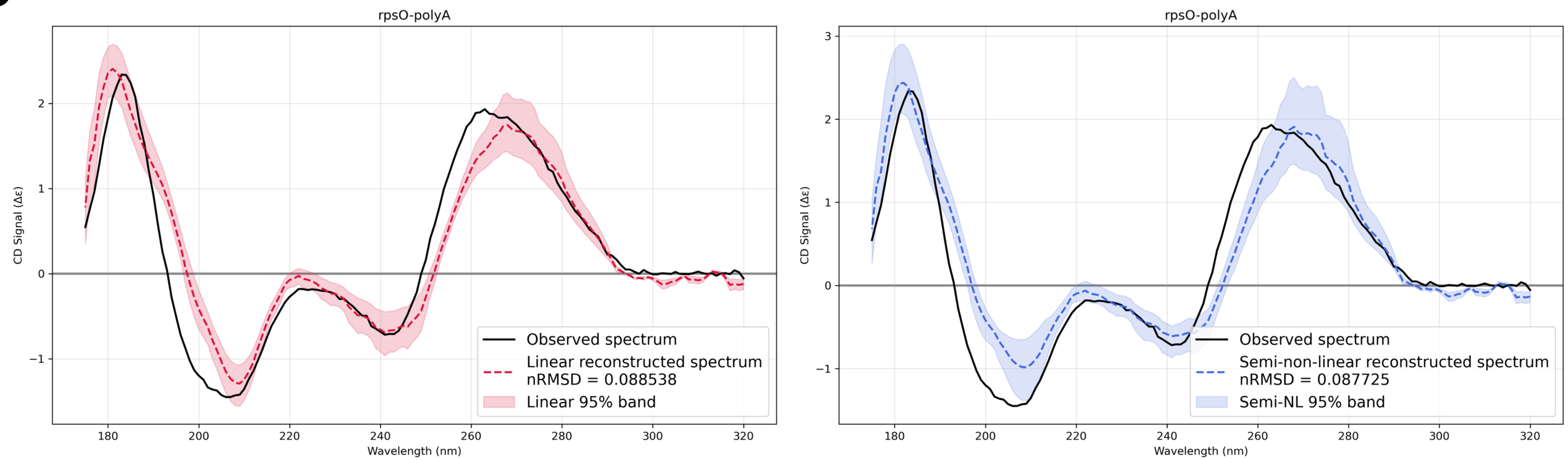

	Disordered RNA	AU rich RNA	Others
Expected	62%	38%	0%
Linear fitting	53.70%	46.30%	0%
Semi-NL fitting	57.74%	42.26%	0%

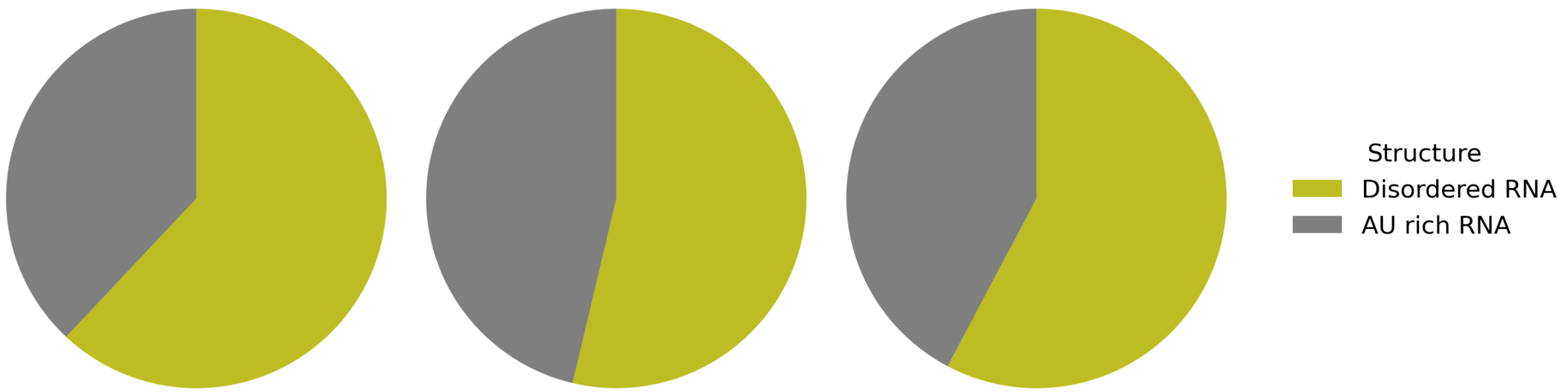

Supplementary Table 1

Supplementary Table 1. Oligonucleotides used to establish spectral references and test NA-CDQuant. References are provided when available. When multiple sequences are shown, each oligonucleotide has been resuspended and then mixed together before annealing. Synthetic sequences were synthetized, provided by Eurogentec, as described under Sample preparation. Literature sequences correspond to spectra extracted from the publication listed.

Spectral reference	Sequence name	Sequence	Reference	Origin
Parallel DNA	1R3X	AGAGAGAA + TTCTCTCT + TTCTCTCT	10.1021/ja973221m	Synthetic
	Parallel DNA	GAAGAAGAAGAAG + CTTCTTCTTCTTC	10.1002/j.1460-2075.1992.tb05463.x	Synthetic
	Triplex Py:Pu-Pu intra 1	TTCTTCTTCTTCTTCTTTTGAAGAAGAAGAAGAATTTTAAGAAGAAGAAGAAG	10.1093/nar/gkh274	Synthetic
	Triplex Py:Pu-Pu intra 2	TTCTTCTTCTTCTTCTTTTGAAGAAGAAGAAGAATTTTAAGAAGAAGAAGAAG	10.1093/nar/gkh274	Synthetic
	Triplex Py:Pu-Py intra 2	CTTCTTCTTCTTCTTTTTTTTCTTCTTCTTCTTCTTTTGAAGAAGAAGAAGAA	ncbi.nlm.nih.gov/books/NBK6545/	Synthetic
Double helix DNA (B-form)	1CS2	CTAAAGCAGTAG + CTACTGCTTTAG	10.1093/nar/27.24.4759	Synthetic
	Br1	TCGTAGCAAGCT + AGCTTGCTACGA		Synthetic
	Br2	GCTCTAGCACCG + CGGTGCTAGACG		Synthetic
	Br3	CCGCTTAAACGC + GCGTTTAAGCGG		Synthetic
	DsrA 22 ADN 10mM	AAGTGCTTCTTGCTTAAGCAAG	10.18388/abp.2014_1941	Synthetic
	B-form-60	E. coli Genomic DNA	10.1002/bip.1979.360180418	Literature
Double helix RNA (A-form)	1RNK	GGCGCAGUGGGCUAGCGCCACUCAAAAGGCCCAU	10.2210/pdb1RNK/pdb	Synthetic
	DsrA 14 ARN	CUUGCUUAAGCAAG	10.18388/abp.2014_1941	Synthetic
	DsrA 22 ARN	AAGUGCUUCUUGCUUAAGCAAG		Synthetic
	Pseudoknot	GGGCUGUUUUUCUCGCUGACUUUCAGCCCCAAACAAAAAAGUCAGCA	10.1016/j.molcel.2005.01.017	Synthetic
	rpsO	CAGAAAAGGGGGCCUGAGUGGCCCCUUUUUUCAA	10.1111/febs.70445	Synthetic
	tRNA	GCGGAUUUAGCUCAGUUGGGAGAGCGCCAGACUGAAGAUCUGGAGGUCCUGUGUUCGAUCCACAGAAUUCGCACCA	10.1038/s41467-019-11356-w	Synthetic
DNA Parallel quadruplex	(G3T)4	GGGTGGGTGGGTGGGT		Synthetic
	2M27	CGGGGCGGGCCTTGGGCGGGGT	10.1093/nar/gkt784	Synthetic
	TT(GGGT)4	TTGGGTGGGTGGGTGGGT		Synthetic
	cMycparallel	TGAGGGTGGGTAGGGTGGGTAA	10.1021/bi048242p	Synthetic
DNA Hybrid quadruplex	2F8U	GGGCGCGGGAGGAATTGGGCGGG	10.1093/nar/gkl610	Synthetic
	2HY9	AAAGGGTTAGGGTTAGGGTTAGGGAA	10.1093/nar/gkm009	Synthetic
	2LOD	GGGATGGGACACAGGGGACGGG	10.1093/nar/gks329	Synthetic
Z-DNA	d(CG)9	CGCGCGCGCGCGCGCGCG	10.1016/j.bmcl.2023.129376	Synthetic
	poly(GC)	(GC)n		Literature
	Z-form	(GC)n	10.1093/nar/13.13.4983	Literature
RNA quadruplex	GGA4	GGAGGAGGAGGA	10.1080/15476286.2025.2512613	Synthetic
	GGU4	GGUGGUGGUGGU	10.1080/15476286.2025.2512613	Synthetic
	RNAquad	UGGCGGGAGGAGGC	10.1093/nar/gkae927	Synthetic
DNA Cruciform prone	1DCW	CCGGTACCGG	10.1073/pnas.97.8.3971	Synthetic
	467D	CCGGGACCGG	10.1038/13277	Synthetic
	ctrl5metC	CCTGGCCAGGCCTGGCCAGG	10.1002/anie.202016822	Synthetic
DNA i-motif	iC3-1	CCCACCCAAACCCACCCA		Synthetic
	iC3-2	CCCACCCAAACCCACCCA		Synthetic
	iC5-1	TCCCCC		Synthetic
	iC5-2	CCCCCAACCCCCTTTTTCCCCCTTCCCCC		Synthetic
	iC5-3	CCCCCTTCCCCCAAAAACCCCCCTTCCCCC		Synthetic
	iC5-4	CCCCCAACCCCCAAAAACCCCCAACCCCC		Synthetic
	i-motif ph5	CCCACCCACCCACCCA	10.1038/363561a0	Synthetic

AU rich RNA	r(A5U5)	(AAAAAUUUUUAAAAAUUUUU)		Synthetic
	rA20rU20	AAAAAAAAAAAAAAAAAAAA + UUUUUUUUUUUUUUUUUUUU		Synthetic
	r(A3U3)	(AAAUUUAAAUUUAAAUUUAAAUUUAAAUUU)		Synthetic
AT rich DNA	(A5T5A5T5)n	(AAAAATTTTTAAAAATTTTTAAAAATTTTTAAAAATTTTTAAAAATTTTTAAAAATTTTTAAAAATTTTT)		Synthetic
	A20(AT)20T20	(AAAAAAAAAAAAAAAAAAAATATATATATATATATATATTTTTTTTTTTTTTTTTTTT)		Synthetic
	d(A3T3)	(AAATTTAAATTTAAATTTAAATTTAAATTT)		Synthetic
	d(A2T2)	(AATTAATTAATTAATTAATTAATTAATT)		Synthetic
	Curved B-DNA Sinden	(CAAAATTTTG)	10.1016/S1067-5701(98)80019-3	Synthetic
A-DNA	A-form-59	C. perfringens Genomic DNA	10.1002/bip.1979.360180418	Literature
	A-form-60	E. coli Genomic DNA		Literature
	A-form-61	M. luteus Genomic DNA		Litteratue
Antiparallel quadruplex 1	148D Lithium defolded	GGTTGGTGTGGTTGG	10.1006/jmbi.1994.1105	Synthetic
	148D	GGTTGGTGTGGTTGG		Synthetic
	TBA Quadruplex	GGTTGGTGTGGTTGG	10.1016/j.ijbiomac.2018.09.102	Synthetic
Antiparallel quadruplex 2	2KM3 Lithium defolded	AGGGCTAGGGCTAGGGCTAGGG	10.1093/nar/gkp630	Synthetic
	2KM3	AGGGCTAGGGCTAGGGCTAGGG		Synthetic
	22CTA	AGGGCTAGGGCTAGGGCTAGGG	10.1093/nar/gkp630	Synthetic
Complex oligonucleotide	7CLS	TTGGATCTGAGAATCAGATGTGGGTGGGTGGGT	10.1093/nar/gkaa738	Synthetic
	8DUT	CTATGTATACAAAGAGGGTGGGTAGGGTGGGTTTAATGCGGCACGC + GCGTGCCGCATTAATTTTTTTTTTTTTTTTTTTTTTGTATACATAG	10.1093/nar/gkad014	Synthetic
	7O5E	CCCGTTTCCTCGCGAAGCATTCGCGCCCGTTTCCT	10.1021/jacs.1c04679	Synthetic
	8S4N	CGCTCACGCCCCGTTTCCCCCCCCCCCGTTTCCCCCGTGAGCG	10.1093/nar/gkae1282	Synthetic
	6R9K	GCGTGGGTCAGGGTGGGTGGGACGC	10.1002/anie.201905372	Synthetic
	6R9L	GCGTGGTTCAGGGTGGGTGGGACGC		Synthetic
	8ABD	TTAGGTGGGTAGGGTGGGCTAGTCATTTTGACTAGG	10.1093/nar/gkac1088	Synthetic
	7PNE	TAGGTGGGTAGGGTGGGCTAGTCATTTTGACTAGG	10.1002/chem.202103718	Synthetic
	4V2S	UUCCGAUGUAGACCCGUCCUCCUUCGCCUGCGUCACGGGUCCUGGUUAGACGCAGGCGUUUUCUG	10.7554/eLife.05375	Synthetic
	rpsO-polyA	CAGAAAAGGGGGCCUGAGUGGCCCCUUUUUUCAAAAAAAAAAAAAAAAA	10.1111/febs.70445	Synthetic

Supplementary Table 2

Supplementary Table 2. Metric comparison of the reliability for the secondary structure estimation between all CD spectrum examples of the validation set. The nRMSD measures the agreement between the experimental and reconstructed CD spectra, while the Pearson correlation coefficient evaluates the relationship between the predicted and expected secondary-structure fractions. Short spectra are derived from the full spectra by truncation of the low wavelength signal to match the wavelength range accessible on conventional CD spectrophotometer.

	Linear				Semi-non-linear			
	Full spectrum (175-320nm)		Short spectrum (200-320nm)		Full spectrum (175-320nm)		Short spectrum (200-320nm)	
	nRMSD	Corr	nRMSD	Corr	nRMSD	Corr	nRMSD	Corr
6R9K	0.038477	0.962972677	0.031645	0.697736019	0.031819	0.945666031	0.021314	0.255118954
6R9L	0.036778	0.959615018	0.30042	0.545942171	0.030042	0.951608243	0.018519	0.333313391
7CLS	0.021184	0.992262425	0.027582	0.993743072	0.021064	0.991099905	0.027443	0.998017023
7O5E	0.058601	0.820064889	0.1293	0.050153127	0.032802	0.912843006	0.064901	0.10325393
7PNE	0.048303	0.958131837	0.043742	0.044608493	0.021735	0.756183497	0.038602	0.441288722
8ABD	0.057779	0.973070706	0.039991	0.001155325	0.053293	0.952222408	0.038018	-0.26517137
8DUT	0.0385	0.922261069	0.027375	0.961727509	0.022697	0.635602684	0.02727	0.927318881
8S4N	0.039099	0.590998728	0.059567	0.341770446	0.031024	0.7749136	0.030399	0.035169011
8R4E	0.031165	0.506339306	0.037857	-0.82563739	0.027481	0.624724412	0.028609	-0.42184925
4V2S	0.034376	0.784187042	0.032723	0.577608722	0.023441	0.992094376	0.028509	0.958397030
rpsO -polyA	0.088538	0.964702822	0.05886	0.974586939	0.087725	0.99130059	0.058541	0.907062498
Mean	0.0448	0.857691502	0.071733	0.396672221	0.034829	0.866205341	0.034737	0.388356256
